# Predictors of chemotherapy-related unplanned acute care in outpatients receiving oral anticancer drugs

**DOI:** 10.1038/s41598-025-18498-6

**Published:** 2025-10-06

**Authors:** Misaki Teramoto, Kenji Kawasumi, Momoka Furuoka, Tsugumichi Sato, Ayako Maeda-Minami, Yuichiro Yamamoto, Yasunari Mano, Toshikatsu Kawasaki, Masashi Nagata, Yohei Kawano

**Affiliations:** 1 Faculty of Pharmaceutical Science, Tokyo University of Science, Tokyo, Japan; 2 Department of Pharmacy, National Cancer Center Hospital East, Chiba, Japan; 3 Department of Pharmacy, Institute of Science Tokyo Hospital, Tokyo, Japan

**Keywords:** Unplanned acute care, Adverse reactions, Outpatient chemotherapy, Oral anticancer drugs, Injectable anticancer drugs, Health care, Health occupations, Medical research, Risk factors

## Abstract

Severe adverse reactions to outpatient chemotherapy receiving oral anticancer drugs often lead to unplanned acute care (UAC) requirements, including unscheduled emergency visits and hospitalizations. These events result in treatment delay or interruption, which reduces the patients’ willingness to continue treatment and negatively impacts their quality of life. The aim of this study was to identify predictive factors for UAC, helping promote safer outpatient chemotherapy through early intervention. A retrospective case-control study was conducted with patients receiving outpatient chemotherapy with oral anticancer drugs at the National Cancer Center Hospital East between April 1, 2020, and March 31, 2021. Patients who experienced UAC were identified as cases, and matched controls were selected from those who did not experience UAC. Conditional multivariate logistic regression analysis was performed to estimate adjusted odds ratios and 95% confidence intervals to examine the association between risk factors and UAC onset. Among cytotoxic drug users, increased blood urea nitrogen concentration and decreased serum sodium concentration were identified as factors associated with increased UAC risk. In molecular-targeted drug users, increased blood creatinine concentration was associated with increased UAC risk. These findings may help optimize patient management and reduce UAC rates in outpatient chemotherapy settings.

**Supplementary Information:**

The online version contains supplementary material available at 10.1038/s41598-025-18498-6.

## Introduction

Cancer care has undergone significant transformation in recent decades, characterized by a growing shift from inpatient to outpatient treatment^1^. This trend has been fueled by multiple factors, including the development of less invasive therapies, improved supportive care, a growing aging population, and rising pressure to reduce healthcare costs^1^.

Outpatient chemotherapy, in particular, has emerged as a cornerstone of modern cancer management, as it enables patients to receive continuous treatment while maintaining some quality of life. Among outpatient chemotherapy approaches, oral anticancer drugs have played a pivotal role in promoting home-based treatment. Their importance has grown in tandem with advancements in supportive care and treatment infrastructure^2^. Compared with injectable therapy, oral chemotherapy can improve patient’s quality of life by eliminating injection-site complications and infusion time constraints, allowing treatment at home^3^. However, the incidence of serious adverse events associated with oral anticancer drug therapy is comparable to that of injectable therapy^4^, indicating that oral administration is not inherently safer.

Safety evaluation of outpatient chemotherapy is typically based on metrics, such as chemotherapy-related hospitalization (CRH) and chemotherapy-related unplanned presentation (CRUP). CRUP progresses to CRH at a very high rate^5^, highlighting the need to reduce the incidence of CRH and CRUP. In this study, these events are collectively referred to as unplanned acute care (UAC). UAC can lead to various issues, including treatment postponement or cancellation, increased healthcare costs, decreased quality of life, and a greater burden on medical staff^5,6^. Preventing the onset of serious adverse events and providing appropriate supportive care early are crucial to reducing the risk of UAC.

To date, only one study^7^ has investigated risk factors associated with UAC in patients experiencing adverse reactions to outpatient anticancer drugs. Another study^8^ has examined predictors of unplanned readmissions in cancer patients, but it did not address chemotherapy-related toxicity as a direct cause of UAC. The landscape of oral anticancer therapy has evolved significantly in the years since those earlier studies were published. Notably, 11, 35, and 16 oral anticancer drugs were approved in Japan in 2000 s, 2010 s, and 2020 alone, respectively. These developments may have altered UAC risk profiles, creating a need for updated evidence to inform clinical decision-making. Therefore, this study aimed to identify clinical and treatment-related factors associated with UAC in patients receiving outpatient chemotherapy, with a specific focus on adverse events induced by anticancer agents. Where applicable, we also sought to determine threshold values for reducing the burden on patients and healthcare providers and improving the safety of outpatient cancer treatment.

## Methods

### Study population

The study included outpatients who received oral anticancer drug therapy (including combination therapy with injectable drug) at the National Cancer Center Hospital East between April 1, 2020, and March 31, 2021. Patients with hematologic malignancies or an unknown performance status (PS) were excluded.

### Study design

A retrospective case-control study based on medical records was conducted to identify factors predicting the occurrence of UAC.

### Identification of cases

Case patients were defined as those who experienced UAC related to oral anticancer drug therapy during the observation period.

### Identification of controls

Eligible control patients were randomly selected from those who did not develop UAC during the study period. A 1:1 to 1:4 matching was performed based on age (± 5 years), sex, treatment regimen, and treatment duration.

### Assessment of outcome

CRH and CRUP due to chemotherapy-induced adverse drug reactions were collectively defined as “UAC” in outpatient chemotherapy. Of these, emergency hospitalization was defined as “adverse drug reactions requiring hospitalization due to anticancer drug therapy,” and unscheduled visits were defined as “unplanned consultations in which the physician determined that hospitalization was unnecessary despite adverse drug reactions.” Causality was determined based on the medical records according to the National Cancer Institute guidelines (February 29, 2012). A five-level classification was used: “not related,” “unlikely,” “possible,” “probable,” and “definite.” Adverse reactions classified as “possible,” “probable,” or “definite” were considered causally related.

### Data collection

The following data were collected: age, sex, cancer type, body mass index (BMI), PS, purpose of treatment (advanced relapse, preoperative or postoperative), line of treatment (first and second or above), type of oral medication, concomitant injection use, and blood laboratory data. For UAC cases and regimens with a course setting, data were obtained at the start of the treatment course during which UAC occurred. For continuous treatment without a course setting, data were collected from the visit date closest to the date of UAC occurrence. For controls, data were obtained at the same time as those for the matched case patients. Missing values were observed in the following laboratory parameters: alkaline phosphatase (ALP) (0.9%), calcium (Ca) (5.9%), chloride (Cl) (0.8%), creatinine (CRE) (0.1%), C-reactive protein (CRP) (3.1%), estimated glomerular filtration rate (eGFR) (0.4%), blood glucose (GLU) (4.6%), lactate dehydrogenase (LDH) (2.0%), lymphocyte (Lymp) (0.7%), monocyte (Mono) (0.8%), neutrophil (Neut) (0.7%), total bilirubin (T-Bil) (0.3%), total protein (TP) (3.5%), blood urea nitrogen (UN) (0.4%), and γ-glutamyl transpeptidase (γ-GTP) (14.6%). As these variables did not follow the normal distribution, we used the median value, which is less sensitive to outliers, for the imputation of missing data.

### Exposure and covariates

As this study was an exploratory investigation, predefining exposure or confounding factors (covariates) was not possible. Therefore, factors identified as predictive of UAC in the analyses were considered exposures, whereas other factors were treated as covariates.

### Statistical analysis

Univariate logistic regression analysis was performed for each variable in the case and control groups. Variables with a *p*-value of < 0.2 in univariate analysis were included in the conditional multivariate logistic regression model as exposure or confounding factors. The adjusted odds ratios, *p*-values, and 95% CIs were calculated. Statistical significance was set at a *p*-value of < 0.05. In this study, variables with a *p*-value < 0.2 in the univariate analysis were included as candidate variables in the multivariable model. This threshold is widely accepted during variable selection to minimize the risk of excluding clinically important confounders. Furthermore, major confounding factors such as age and sex were controlled through matching, which helped reduce potential bias and multicollinearity.

Multicollinearity among explanatory variables was defined as a variance inflation factor of ≥ 10. Model goodness of fit was assessed using the Hosmer–Lemeshow test, and predictive accuracy was assessed using the area under the receiver operating characteristic curve.

Secondary analyses: patients were classified into substandard, standard, and overstandard groups based on laboratory parameters identified as predictive factors for UAC. Odds ratios and 95% CIs were estimated for these groups.

All statistical analyses were performed using IBM SPSS Statistics version 23.0; (SPSS Inc., Armonk, NY, USA).

### Ethical considerations

This study was approved by the Ethics Review Committee of the National Cancer Center Hospital East (approval number: 2022 − 113), which also granted the waiver of individual informed consent. The Ethics Review Committee of the Tokyo University of Science approved the joint ethical review process (approval number: 23034).

As this was a retrospective study that did not involve any invasive procedures, interventions, or the use of human-derived specimens, and was based on anonymized data, the requirement for obtaining individual informed consent was waived in accordance with the Ethical Guidelines for Medical and Health Research Involving Human Subjects enacted by the Japanese government.

Instead, information about the study was made publicly available via institutional websites and/or on-site postings to provide patients with the opportunity to opt out.

All methods were performed in accordance with the relevant guidelines and regulations.

## Results

### Study participants

**Fig. 1:**
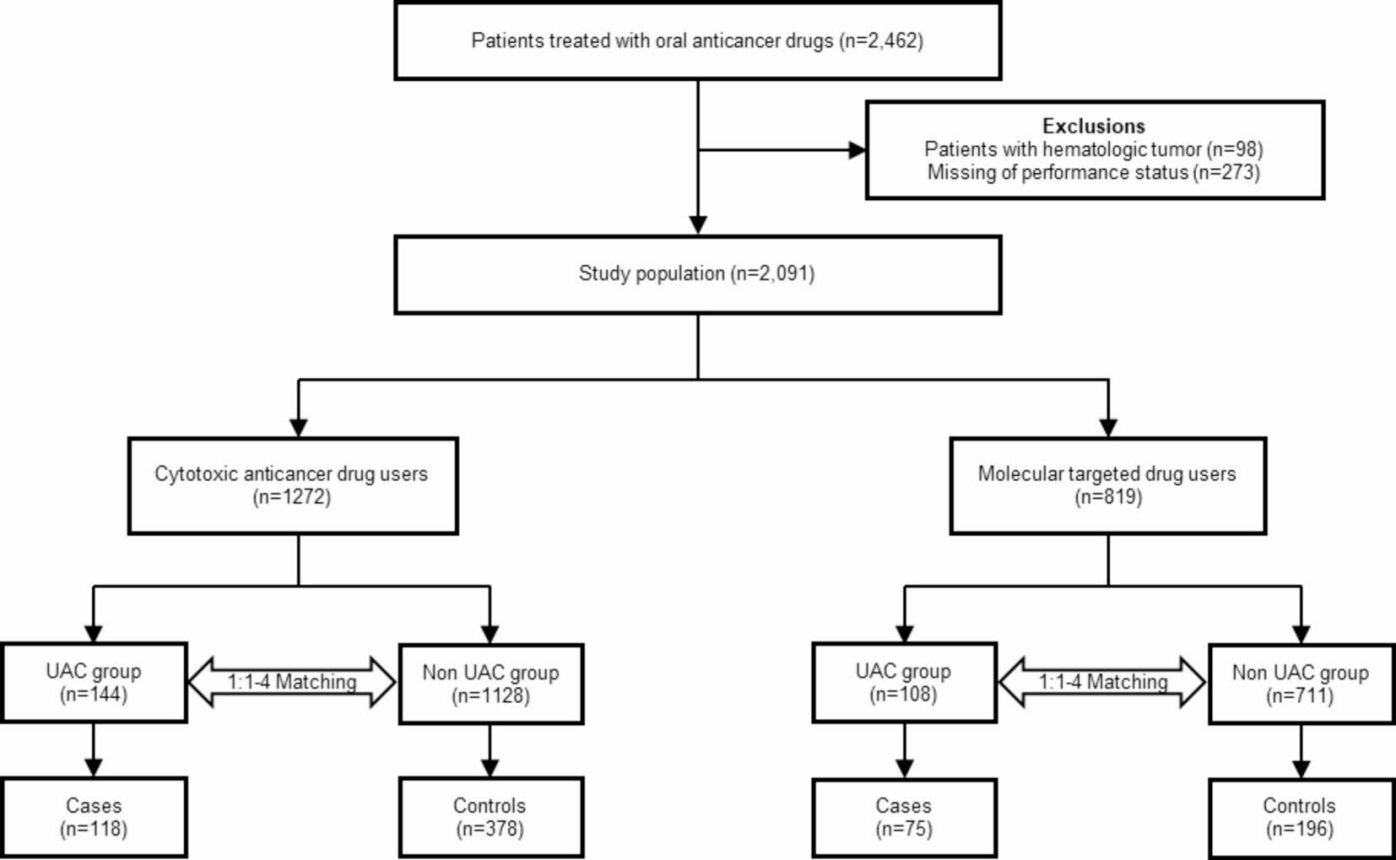
Flowchart for the Selection of Cases and Controls. UAC: unplanned acute care.

Among 2,462 participants, 98 patients with hematologic malignancies and 273 with unknow PS were excluded, resulting in a final study population of 2,091 patients. Using this dataset, 252 case patients with chemotherapy-related UAC during the observation period and 1,839 possible control patients without UAC were identified. As a result of 1:1 to 1:4 matching, in total 193 case patients were matched with 574 controls. Among cytotoxic drug users, 118 case patients and 378 control patients were selected. Among molecular-targeted drug users, 75 case patients and 196 control patients were selected (Fig. 1).

### Characteristics of cases and controls

Among cytotoxic drug users, no significant differences were observed in patient background characteristics between the case and control groups. However, significant differences were found in 10 blood laboratory parameters: TP, albumin (ALB), UN, Na, Ca, CRP, red blood cell (RBC), hemoglobin (Hb), platelet (Plt), and lymphocyte (Lymp) levels (Table 1).

**Table 1:** Characteristics and laboratory values of case and control patients among cytotoxic drug users (Univariate Analysis).

Characteristics	Case group (*n* = 118)		Control group (*n* = 378)		Odds ratio (95% CI)	*p*-value	Laboratory variables	Case group (*n* = 118)	Control group (*n* = 378)	Odds ratio (95% CI)	*p*-value
	*n*	%	*n*	%							
Age (mean ± SD)	66.3 ± 9.4		67.1 ± 8.5		0.99 (0.97–1.01)	0.42	TP (g/dL)	6.5 ± 0.6	6.7 ± 0.6	0.53 (0.37–0.77)	< 0.01*
Sex							ALB (g/dL)	3.6 ± 0.5	3.8 ± 0.5	0.47 (0.31–0.70)	< 0.01*
Male	73	61.9	241	63.8	1.00 (ref)	0.71	GLU (mg/dL)	129.2 ± 42.9	124.6 ± 47.3	1.00 (0.99–1.01)	0.35
Female	45	38.1	137	36.2	1.08 (0.71–1.66)		UN (mg/dL)	17.0 ± 8.7	14.5 ± 4.5	1.07 (1.03–1.11)	< 0.01*
BMI (mean ± SD)	21.2 ± 3.8		21.7 ± 3.5		0.96 (0.91–1.02)	0.23	CRE (mg/dL)	0.8 ± 0.3	0.8 ± 0.2	2.16 (0.92–5.06)	0.08†
Cancer Type							eGFR (mL/min/1.73 m²)	69.8 ± 18.5	74 ± 21.7	0.99 (0.98–1.00)	0.06†
Lung cancer	5	4.2	24	6.3	1.00 (ref)		Na (mmol/L)	139.9 ± 3.5	141.2 ± 2.8	0.88 (0.82–0.94)	< 0.01*
Colorectal cancer	41	34.7	143	37.8	1.38 (0.49–3.83)	0.54	K (mmol/L)	4.1 ± 0.4	4.1 ± 0.4	1.17 (0.73–1.88)	0.51
Gastric cancer	25	21.2	70	18.5	1.71 (0.59–4.98)	0.32	Cl (mmol/L)	103.8 ± 3.6	104.3 ± 3.2	0.96 (0.90–1.02)	0.18†
Pancreatic cancer	26	22.0	75	19.8	1.66 (0.58–4.81)	0.35	Ca (mg/dL)	8.8 ± 0.5	8.9 ± 0.4	0.41 (0.25–0.68)	< 0.01*
Breast cancer	8	6.8	25	6.6	1.54 (0.44–5.36)	0.50	AST (U/L)	26.7 ± 27.2	27 ± 16.9	0.99 (0.99–1.01)	0.90
Other	13	11.0	41	10.8	1.52 (0.48–4.80)	0.47	ALT (U/L)	19.4 ± 18.8	20.6 ± 14.2	0.99 (0.98–1.01)	0.45
Performance Status							γ–GTP (U/L)	80.9 ± 208.2	58.2 ± 92.8	1.00 (1.00–1.00)	0.13†
0	80	67.8	250	66.1	1.00 (ref)		T-Bil (mg/dL)	0.7 ± 0.4	0.7 ± 0.3	1.26 (0.73–2.17)	0.40
1	32	27.1	116	30.7	0.862 (0.54–1.37)	0.53	ALP (U/L)	363.5 ± 638.3	280.4 ± 183.5	1.00 (1.00–1.00)	0.05†
2	6	5.1	10	2.6	1.87 (0.66–5.32)	0.24	LDH (U/L)	231.4 ± 191.8	209.5 ± 132.4	1.00 (1.00–1.00)	0.19†
3	-	-	2	0.5	-	-	CRP (mg/dL)	1.5 ± 3.0	0.8 ± 2.0	1.12 (1.03–1.22)	0.01*
4	-	-	-	-	-	-	RBC (10 ^4^/***µ*** L)	356.3 ± 72.2	369.8 ± 62.0	0.99 (0.99–0.99)	0.04*
Treatment setting							Hb (g/dL)	11.0 ± 2.1	11.5 ± 1.8	0.85 (0.76–0.95)	< 0.01*
Progression/recurrence	74	62.7	248	65.6	1.00 (ref)	0.57	Plt (10 ^4^/***µ*** L)	18.8 ± 6.9	20.5 ± 8.6	0.97 (0.95–0.99)	0.04*
Cure (adjuvant/neoadjuvant)	44	37.3	130	34.4	1.13 (0.74–1.74)		WBC (10 ^2^/***µ*** L)	49.2 ± 20.1	51 ± 27.1	0.99 (0.99–1.01)	0.53
Treatment line							Neut (10 ^2^/***µ*** L)	32.5 ± 19.6	31.8 ± 22.7	0.99 (0.99–1.01)	0.76
1st	58	49.2	182	48.1	1.00 (ref)	0.85	Lymp (10 ^2^/***µ*** L)	11.5 ± 5.0	13.1 ± 5.3	0.94 (0.90–0.98)	< 0.01*
≧ 2nd	60	50.8	196	51.9	0.96 (0.64–1.45)		Mono (10 ^2^/***µ*** L)	3.5 ± 2.0	3.9 ± 2.0	0.91 (0.81–1.01)	0.08†
Treatment classification											
Monotherapy	48	40.7	158	41.8	1.00 (ref)	0.83					
Injection combination therapy	70	59.3	220	58.2	1.05 (0.69–1.59)						

Crude odds ratios for continuous variables indicate association with 1-unit increase.

†*p* < 0.2, *p < 0.05.

UAC, unplanned acute care; BMI: Body mass index; TP: total protein; ALB: albumin; GLU: blood glucose; UN: blood urea nitrogen; CRE: creatinine; eGFR: estimated glomerular filtration rate; AST: aspartate aminotransferase; ALT: alanine aminotransferase; γ-GTP: γ-glutamyl transpeptidase; T-Bil: total bilirubin; ALP: alkaline phosphatase; LDH: lactate dehydrogenase; CRP: C-reactive protein; RBC: red blood cell; Hb: hemoglobin; Plt: platelet; WBC: white blood cell; Neut: neutrophil; Lymp: lymphocyte; Mono: monocyte.

Among molecular-targeted drug users, no significant differences in patient background were observed; however, ALB, CRE, and CRP levels differed significantly (Table 2).

**Table 2:** Characteristics and laboratory values of case and control patients among Molecular-targeted drug users (Univariate Analysis).

Characteristics	Case group (*n* = 75)		Control group (*n* = 196)		Odds ratio (95% CI)	*p*-value	Laboratory variables	Case group (*n* = 75)	Control group (*n* = 196)	Odds ratio (95% CI)	*p*-value
	*n*	%	*n*	%							
Age (mean ± SD)	67.1 ± 10.5		67.8 ± 9.4		0.99 (0.97–1.02)	0.59	TP (g/dL)	6.8 ± 0.6	6.9 ± 0.5	0.77 (0.47–1.27)	0.31
Sex							ALB (g/dL)	3.7 ± 0.5	3.9 ± 0.4	0.51 (0.28–0.92)	0.03*
Male	26	34.7	70	35.7	1.00 (ref)	0.87	GLU (mg/dL)	119.7 ± 36.3	112.6 ± 30.5	1.01 (0.99–1.01)	0.11†
Female	49	65.3	126	64.3	1.05 (0.6–1.83)		UN (mg/dL)	16.6 ± 8.3	15.6 ± 4.6	1.03 (0.98–1.07)	0.22
BMI (mean ± SD)	22.6 ± 4.1		22.4 ± 3.7		1.02 (0.95–1.09)	0.60	CRE (mg/dL)	0.9 ± 0.4	0.8 ± 0.2	2.57 (1.06–6.25)	0.04*
Cancer Type							eGFR (mL/min/1.73 m²)	62.3 ± 20.7	64.6 ± 17.2	0.99 (0.98–1.01)	0.35
Lung cancer	38	50.7	106	54.1	1.00 (ref)		Na (mmol/L)	141.0 ± 2.9	141.4 ± 2.7	0.96 (0.87–1.06)	0.40
Colorectal cancer	6	8.0	20	10.2	0.84 (0.31–2.24)	0.72	K (mmol/L)	4.1 ± 0.4	4.1 ± 0.3	1.16 (0.56–2.43)	0.69
Breast cancer	12	16.0	25	12.8	1.34 (0.61–2.93)	0.46	Cl (mmol/L)	104.5 ± 2.8	104.7 ± 3.2	0.99 (0.91–1.08)	0.77
Other	19	25.3	45	23.0	1.18 (0.61–2.26)	0.62	Ca (mg/dL)	8.9 ± 0.5	9.0 ± 0.4	0.63 (0.35–1.15)	0.13†
Performance Status							AST (U/L)	42.1 ± 89.1	27.6 ± 14.3	1.01 (0.99–1.03)	0.07†
0	35	46.7	96	49.0	1.00 (ref)		ALT (U/L)	42.4 ± 140.1	22.3 ± 20.9	1.01 (0.99–1.02)	0.18†
1	32	42.7	84	42.9	1.04 (0.56–1.83)	0.88	γ–GTP (U/L)	54.8 ± 97.6	49.7 ± 74.7	1.00 (0.99–1.00)	0.65
2	8	10.7	25	7.7	1.46 (0.57–3.75)	0.43	T-Bil (mg/dL)	0.7 ± 0.5	0.7 ± 0.4	1.25 (0.67–2.31)	0.48
3	-	-	1	0.5	-	-	ALP (U/L)	278.6 ± 253.8	246.4 ± 160.4	1.00 (0.99–1.00)	0.23
4	-	-	-	-	-	-	LDH (U/L)	261.1 ± 131.6	230.2 ± 131.5	1.00 (1.00–1.00)	0.11†
Treatment setting							CRP (mg/dL)	1.6 ± 4.3	0.6 ± 1.2	1.16 (1.02–1.32)	0.02*
Progression/recurrence	74	98.7	195	99.5	1.00 (ref)	0.50	RBC (10 ^4^/***µ*** L)	393.5 ± 77.6	401.5 ± 61.4	0.99 (0.99–1.00)	0.38
Cure (adjuvant/neoadjuvant)	1	1.3	1	0.5	2.64 (0.16–42.70)		Hb (g/dL)	12.0 ± 2.0	12.4 ± 1.7	0.88 (0.76–1.03)	0.10†
Treatment line							Plt (10 ^4^/***µ*** L)	20.2 ± 9.2	18.4 ± 6.3	1.03 (0.99–1.07)	0.07†
1st	35	46.7	68	34.7	1.00 (ref)	0.07†	WBC (10 ^2^/***µ*** L)	54.0 ± 23.7	49.9 ± 18.3	1.01 (0.99–1.02)	0.13†
** ≥** 2nd	40	53.3	128	65.3	0.61 (0.35–1.04)		Neut (10 ^2^/***µ*** L)	35.7 ± 19.6	32.1 ± 15.6	1.01 (0.99–1.03)	0.12†
Treatment classification							Lymp (10 ^2^/***µ*** L)	12.4 ± 5.8	12.6 ± 5.1	0.99 (0.95–1.05)	0.85
Monotherapy	70	93.3	183	93.4	1.00 (ref)	0.99	Mono (10 ^2^/***µ*** L)	3.9 ± 2.1	3.5 ± 1.5	1.14 (0.98–1.33)	0.09†
Injection combination therapy	5	6.7	13	6.6	1.01 (0.35–2.92)						

Crude odds ratios for continuous variables indicate an association with a 1-unit increase.

**†***p* < 0.2, *p < 0.05.

UAC: Unplanned acute care; BMI: Body mass index; TP: total protein; ALB: albumin; GLU: blood glucose; UN: blood urea nitrogen; CRE: creatinine; eGFR: estimated glomerular filtration rate; AST: aspartate aminotransferase; ALT: alanine aminotransferase; γ-GTP: γ-glutamyl transpeptidase; T-Bil: total bilirubin; ALP: alkaline phosphatase; LDH: lactate dehydrogenase; CRP: C-reactive protein; RBC: red blood cell; Hb: hemoglobin; Plt: platelet; WBC: white blood cell; Neut: neutrophil; Lymp: lymphocyte; Mono: monocyte.

### Analysis of UAC occurrence predictors

Among cytotoxic drug users, univariate logistic regression analysis between cases and controls identified 17 variables with *p* < 0.2: TP, ALB, UN, CRE, eGFR, Na, Cl, Ca, γ-GTP, ALP, LDH, CRP, RBC, Hb, Plt, Lymp, and Mono levels (Table 1). Conditional multivariate logistic regression analysis using these variables as covariates showed that blood UN concentration (adjusted odds ratio: 1.05, 95% confidence interval [CI]: 1.00–1.10, *p*-value: 0.035) and serum Na concentration (adjusted odds ratio 0.83, 95% CI: 0.73–0.95, *p*-value: 0.006) were associated with UAC (Table 3).

**Table 3:** Factors associated with UAC at the start of treatment in cytotoxic drug users (Multivariate Analysis).

Factor	Adjusted odds ratio (95% CI)	*p*-value
TP	0.72 (0.43–1.22)	0.22
ALB	0.90 (0.42–1.91)	0.78
UN	1.05 (1.00–1.10)	0.04*
CRE	0.43 (0.08–2.25)	0.31
eGFR	0.99 (0.97–1.00)	0.14
Na	0.83 (0.73–0.95)	0.01*
Cl	1.10 (0.98–1.23)	0.12
Ca	0.71 (0.33–1.51)	0.38
γ-GTP	1.00 (0.99–1.00)	0.85
ALP	1.00 (1.00–1.00)	0.49
LDH	1.00 (0.99–1.00)	0.76
CRP	1.05 (0.94–1.18)	0.40
RBC	1.01 (0.99–1.01)	0.15
Hb	0.87 (0.67–1.13)	0.30
Plt	0.97 (0.94–1.00)	0.08
Lymp	0.99 (0.94–1.04)	0.70
Mono	0.92 (0.81–1.04)	0.19

Adjusted for all listed covariates. The Hosmer–Lemeshow test yielded a *p*-value of 0.893, indicating a good model fit. AUC was 0.703. Additionally, the variance inflation factors (VIFs) for all variables were below 10, indicating no evidence of multicollinearity.

Adjusted odds ratios for continuous variables indicate an association with a 1-unit increase.

*: *p* < 0.05; UAC: unplanned acute care.

For molecular-targeted drug users, univariate logistic regression analysis identified 14 variables with a *p*-value of < 0.2: treatment line, ALB, glucose, CRE, Ca, aspartate aminotransferase, alanine aminotransferase, LDH, CRP, Hb, Plt, white blood cell, neutrophil, and Mono levels (Table 2).

Conditional multivariate logistic regression analysis using these variables as covariates showed that the treatment line (adjusted odds ratio: 0.53, 95% CI: 0.29–0.97, *p*-value: 0.040) and CRE levels (adjusted odds ratio: 3.07, 95% CI: 1.14–8.27, *p*-value: 0.027) were significantly associated with UAC (Table 4).

**Table 4:** Factors associated with UAC at the start of treatment in Molecular-targeted drug users (Multivariate Analysis).

Factor	Adjusted odds ratio (95% CI)	*p*-value
Treatment Line	0.53 (0.29–0.97)	0.04*
ALB	1.04 (0.42–2.60)	0.93
GLU	1.00 (0.99–1.01)	0.47
CRE	3.07 (1.14–8.27)	0.03*
Ca	0.85 (0.37–1.94)	0.70
AST	1.02 (0.99–1.04)	0.20
ALT	1.00 (0.98–1.02)	0.94
LDH	0.99 (0.99–1.00)	0.59
CRP	1.12 (0.96–1.31)	0.14
Hb	0.84 (0.70–1.01)	0.07
Plt	1.04 (0.99–1.10)	0.07
WBC	1.01 (0.95–1.06)	0.84
Neut	0.99 (0.93–1.05)	0.73
Mono	1.06 (0.85–1.34)	0.59

Adjusted for all listed covariates. The Hosmer–Lemeshow test yielded a *p*-value of 0.973, indicating a good model fit, but AUC was 0.681, suggesting limited predictive accuracy. Additionally, the variance inflation factors (VIFs) for all variables were below 10, indicating no evidence of multicollinearity.

Adjusted odds ratios for continuous variables indicate an association with a 1-unit increase.

*: *p* < 0.05.

UAC: unplanned acute care.

### Types and frequencies of adverse drug reactions in case patients

The most frequent adverse reactions in cytotoxic drug users were anorexia (22.0%), fever (19.5%), nausea and vomiting (19.5%), diarrhea (18.6%), and skin problems (9.3%) (Table 5). The most frequent adverse reactions among molecular-targeted drug users included skin problems (20.0%), fever (17.3%), pneumonia (10.7%), nausea and vomiting (9.3%), and diarrhea (8.0%). Compared to a previous study conducted in 2015^7^, the types and frequencies of adverse drug reactions among molecular-targeted drug users have changed (Table 5). Specifically, the rates of anorexia, nausea and vomiting, and diarrhea decreased from 20 to 6.7%, 16.7–9.3%, and 36.7–8.0%, respectively. The incidence of dehydration decreased from 10 to 2.7%, and that of skin disorders decreased from 56.7 to 20.0%.

**Table 5:** Comparison of adverse reactions leading to UAC in 2020 and 2015.

Adverse reaction	2020						2015					
	Cytotoxic anticancer drug user (*n* = 118)		Molecular-targeted drug users (*n* = 75)		All (*n* = 193)		Cytotoxic anticancer drug users (*n* = 65)		Molecular-targeted drug users (*n* = 30)		All (*n* = 95)	
	*n*	%	*n*	%	*n*	%	*n*	%	*n*	%	*n*	%
Fever	23	19.5	13	17.3	36	18.7	19	29.2	6	20.0	25	26.3
Anorexia	26	22.0	5	6.7	31	16.1	14	21.5	6	20.0	20	21.1
Nausea/Vomiting	23	19.5	7	9.3	30	15.5	12	18.5	5	16.7	17	17.9
Diarrhea	22	18.6	6	8.0	28	14.5	21	32.3	11	36.7	32	33.7
Skin reaction	11	9.3	15	20.0	26	13.5	11	16.9	17	56.7	28	29.5
Stomachache	10	8.5	1	1.3	11	5.7	5	7.7	3	10.0	8	8.4
Fatigue	7	5.9	4	5.3	11	5.7	-	-	-	-	-	-
Stomatitis	9	7.6	2	2.7	11	5.7	6	9.2	1	3.3	7	7.4
Pneumonia	3	2.5	8	10.7	11	5.7	3	4.6	2	6.7	5	5.3
Dehydration	7	5.9	2	2.7	9	4.7	3	4.6	3	10.0	6	6.3
FN	8	6.8	1	1.3	9	4.7	-	-	-	-	-	-
Respiratory symptoms	1	0.8	6	8.0	7	3.6	-	-	-	-	-	-
Pain	2	1.7	4	5.3	6	3.1	-	-	-	-	-	-
Enteritis	5	4.2	0	0.0	5	2.6	3	4.6	0	0.0	3	3.2
Constipation	4	3.4	0	0.0	4	2.1	1	1.5	2	6.7	3	3.2
Cellulitis	1	0.8	3	4.0	4	2.1	-	-	-	-	-	-
Edema	2	1.7	2	2.7	4	2.1	2	3.1	1	3.3	3	3.2
IR	4	3.4	0	0.0	4	2.1	-	-	-	-	-	-
Dizziness	2	1.7	1	1.3	3	1.6	4	6.2	0	0.0	4	4.2
Light-headedness	1	0.8	2	2.7	3	1.6	-	-	-	-	-	-
Weakness	1	0.8	2	2.7	3	1.6	-	-	-	-	-	-
Bleeding	1	0.8	2	2.7	3	1.6	-	-	-	-	-	-
Liver disorder	1	0.8	2	2.7	3	1.6	2	3.1	1	3.3	3	3.2
Kidney disorder	2	1.7	1	1.3	3	1.6	-	-	-	-	-	-
Cytopenia	-	-	-	-	-	-	2	3.1	1	3.3	3	3.2
Epigastralgia	-	-	-	-	-	-	2	3.1	1	3.3	3	3.2
Others*	25	21.2	18	24.0	43	22.3	20	30.8	29	96.7	49	51.6

*Adverse reactions listed as “Others” represent cases with fewer than three occurrences.

The number of occurrences of each adverse reaction is shown as an overlapping number in cases where a combination of multiple adverse reactions led to UAC.

UAC: unplanned acute care.

### Comparison of UAC risk based on the reference ranges for test values

**Fig. 2:**
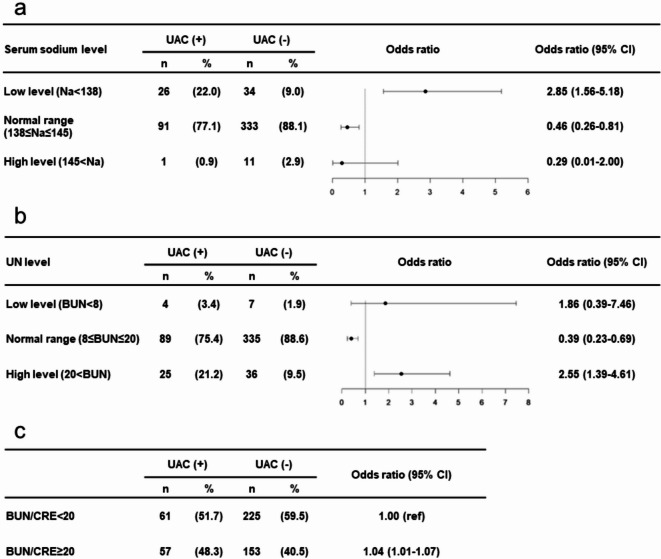
Forest Plot Showing the Results of a Secondary Analysis in Cytotoxic Drug Users. (**a**) Serum Sodium Levels (**b**) BUN Levels. Serum sodium concentrations were categorized into three groups based on reference values: “Low level” (<138 mmol/L), “Normal range” (138–145 mmol/L), and “High level” (>145 mmol/L). BUN levels were categorized into three groups based on clinical reference values: low (<8 mg/dL), normal (8–20 mg/dL), and high (>20 mg/dL). Crude odds ratios for the occurrence of UAC were calculated by comparing each group to a combination of the other two. The comparisons were as follows: “Low level” vs. (“Normal range” + “High level”),“Normal range” vs. (“Low level” + “High level”), and “High level” vs. (“Low level” + “Normal range”). UAC, unplanned acute care; CI, confidence interval; BUN, blood urea nitrogen. (**c**) BUN/CRE Ratio The BUN/CRE ratio, an indicator of dehydration, was analyzed to assess its association with the occurrence of UAC in cytotoxic drug users. Ratios were divided into two categories: BUN/CRE<20 and BUN/CRE ≥20. Crude odds ratios were calculated to compare the risk of UAC between the two groups. UAC, unplanned acute care; CI, confidence interval; BUN, blood urea nitrogen; CRE, creatinine.

In cytotoxic drug users, the low sodium group (Na < 138 mmol/L) resulted in an increased risk of UAC (odds ratio: 2.85, 95% CI: 1.56–5.18, *p-*value: <0.001) (Fig. 2-a). Additionally, in cytotoxic drug users, the high UN group (UN > 20 mg/dL) showed an increased risk of UAC (odds ratio: 2.55, 95% CI: 1.39–4.61, *p*-value: 0.002) (Fig. 2-b). In cytotoxic drug users, the BUN/CRE ≥ 20 group, an indicator of dehydration, was significantly associated with UAC (odds ratio: 1.04, 95% CI: 1.01–1.07, *p*-value: 0.009) (Fig. 2-c).

**Fig. 3:**
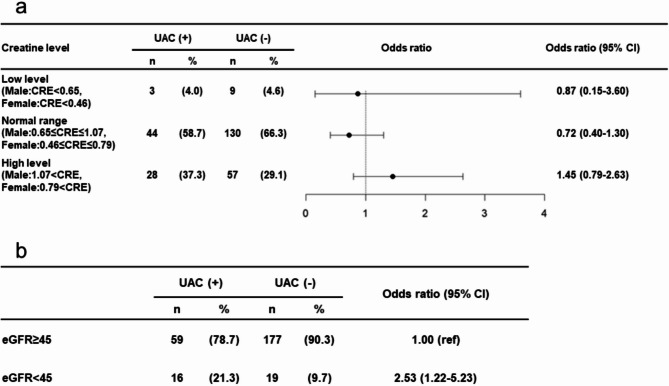
Forest Plot Showing the Results of a Secondary Analysis among Molecular-targeted Drug Users. (a) CRE. Blood creatinine levels were categorized into three groups based on sex-specific reference values: “Low level” (men: CRE < 0.65 mg/dL, women: CRE < 0.46 mg/dL), “Normal range” (men: 0.65 ≤ CRE ≤ 1.07 mg/dL, women: 0.46 ≤ CRE ≤ 0.79 mg/dL), and “High level” (men: CRE > 1.07 mg/dL, women: CRE > 0.79 mg/dL). Crude odds ratios for the occurrence of UAC were calculated by comparing each group to a combination of the other two. (b) eGFR. Patients were divided into two groups based on eGFR levels: <45 mL/min/1.73 m² (more than moderate renal function decline) and ≥ 45 mL/min/1.73 m² (less than moderate renal function decline). Crude odds ratios for the occurrence of UAC were calculated for these categories. UAC, unplanned acute care; CI, confidence interval; CRE, creatinine; eGFR, estimated glomerular filtration rate.

In molecular-targeted drug users, no significant differences in UAC incidence were found among the groups classified according to blood CRE concentrations (Fig. 3-a). In molecular-targeted drug users, the eGFR of < 45 mL/min/1.73 m^2^ group showed an increased risk of UAC (odds ratio: 2.53, 95% CI: 1.22–5.23, *p*-value: 0.011) (Fig. 3-b).

## Discussion

This study identified distinct laboratory risk factors for UAC among patients undergoing outpatient chemotherapy with oral anticancer agents. Importantly, the predictive value of serum sodium and BUN concentrations in cytotoxic drug users, as well as eGFR in molecular-targeted drug users, offers practical clinical markers for early intervention.

The association between decreased sodium concentrations and increased BUN in patients treated with cytotoxic agents likely reflects dehydration, a common consequence of chemotherapy-induced vomiting, diarrhea, and anorexia^5,9–12^. Previous studies have established that fluid loss through these symptoms can lead to prerenal azotemia, characterized by elevated BUN levels due to increased tubular reabsorption^13^. According to the FDA prescribing information, capecitabine treatment should be discontinued and appropriate rehydration administered if Grade 2 or higher dehydration is observed, highlighting the critical importance of managing fluid loss during oral chemotherapy^14^. This regulatory emphasis is echoed in the EMA-approved Summary of Product Characteristics for S-1 (tegafur/gimeracil/oteracil), which advises treatment interruption until dehydration is fully resolved, and its underlying cause addressed^15^. Moreover, chemotherapy-induced diarrhea can result in fatal outcomes due to dehydration and electrolyte imbalances, which further increase the risk of severe adverse events^16^. These observations are consistent with the results of our study. Our findings build on this understanding by suggesting that even mild reductions in sodium (< 138 mmol/L) and moderate elevations in BUN (> 20 mg/dL) may signal a risk for UAC, warranting closer monitoring and supportive care. Clinical interventions such as oral rehydration, antiemetics, and dietary guidance may help prevent escalation to emergency care.

In patients receiving molecular-targeted drug therapy, elevated blood CRE levels were significantly associated with the occurrence of UAC in primary analysis. However, no clear clinically meaningful cut-off value could be identified. Therefore, a secondary analysis was performed using eGFR, a more comprehensive measure of renal function. This analysis evidenced that patients with eGFR of < 45 mL/min/1.73 m^2^ had a significantly higher risk of UAC.

Osimertinib, taken by five patients with UAC and eGFR < 45 mL/min/1.73 m^2^ in this study, is commonly used to treat lung cancer without dose reduction due to renal function. However, the AUC of its metabolite, AZD5104, increases in cases of moderate renal dysfunction (30 mL/min/1.73 m^2^ ≤ eGFR < 50 mL/min/1.73 m^2^), and this can lead to an increase in Grade 2 or higher adverse drug reactions^17^.

In addition to osimertinib^18^, other molecular-targeted drugs that were associated with UAC and decreased eGFR values were the substrates of breast cancer resistance protein (BCRP) and P-glycoprotein (P-gp), including palbociclib (two patients)^19,20^, afatinib (two patients)^21^, lenvatinib (one patient)^22^, regorafenib (one patient)^23^, abemaciclib (one patient)^18^, and sunitinib (one patient)^24,25^. Furthermore, uremic toxins such as kynurenic acid, hippuric acid, and indoxyl sulfate, which accumulate in the body during renal dysfunction, inhibit BCRP^26^. Notably, the risk of presenting with elevated CRE levels, linked to UAC occurrence in this study, was positively correlated with indoxyl sulfate levels^27^. Additionally, everolimus (one patient)^28^, and M-4, a metabolite of alectinib (one patient)^29^, are substrates of P-gp, which is involved in active drug transport. P-gp is inhibited by endothelin-1, a uremic substance whose production increases during renal injury^30^. Although renal dysfunction is not believed to cause significant changes in the pharmacokinetics of the unchanged forms of these molecularly targeted drugs^20,27,31–34^, the excretion rate of structurally similar metabolites, such as osimertinib, may be reduced. This may lead to increased blood metabolite concentrations and a high risk of adverse drug reactions in patients with renal dysfunction.

Furthermore, all these drugs are metabolized by CYP3A4, an enzyme inhibited by uremic toxins, such as benzyl alcohol, *p*-cresol, indoxyl sulfate, and hippuric acid^35^. Thus, the inhibition of BCRP, P-gp, and CYP3A4 by uremic toxins in the case patients in this study may have contributed to severe adverse drug reactions.

At the start of the regimen course or during regular visits, patients with eGFR of < 45 mL/min/1.73 m^2^ or a tendency toward decreased renal function should be carefully monitored for adverse drug reactions. Interventions by healthcare professionals, such as encouraging adequate hydration and adjusting concomitant medications that may cause renal dysfunction, are crucial for preventing serious complications.

Among patients receiving molecular-targeted drug therapy, those receiving first-line therapy were at a higher risk of UAC than those receiving second-line or later therapy. Treatment line was included as a covariate in the multivariable model; however, we interpreted this association as reflecting appropriate clinical adjustments—such as dose reductions or regimen changes following adverse reactions during first-line therapy—rather than intrinsic patient susceptibility. Therefore, although treatment line showed statistically significant effects, we did not consider it a clinically meaningful independent predictor of UAC risk.

The breakdown of adverse events observed herein differed from that reported in the 2015 study^7^, particularly in the molecular-targeted drug group, likely due to changes in the drugs used over time (Supplemental Table S1). That earlier research also identified low BMI (< 18.5 kg/m²) at baseline and throughout treatment as a significant predictor of UAC occurrence. In contrast, no significant association between low BMI and an increased UAC incidence was found in the present study (Supplemental Figure S1). While a trend toward a higher incidence of UAC was noted among patients with low BMI receiving cytotoxic drugs, this association was not observed in those treated with molecular-targeted agents.

This difference may be due to changes in the drugs used over time. In a study in 2015, gefitinib and afatinib were the predominant EGFR-TKIs^36^, whereas in 2020, when the data for the current study were collected, osimertinib was predominantly used^37^. Osimertinib, which was approved as a second-line treatment in 2015 and first-line treatment in 2018, is associated with reduced adverse drug reaction incidence, including that of skin disorders and gastrointestinal toxicity. These drug changes may have reduced the frequency of UAC in patients with low BMI. However, frequent adverse drug reactions have been reported in underweight patients (< 45 kg) taking osimertinib^17^. Therefore, regardless of the drug type, careful monitoring of patients with low BMI is warranted in future clinical practice.

Our findings have implications for clinical risk stratification. The developed model, with AUCs of 0.703 and 0.681, falls within accepted ranges for clinical prediction tools. While moderate in discrimination, such models remain useful when paired with flexible decision thresholds. Clinicians can calibrate these thresholds depending on their clinical priorities—for instance, emphasizing sensitivity to detect patients at high-risk early, or specificity to reduce overtreatment—thereby enhancing the model’s practical value^38^.

This study has some limitations. First, the single-center nature of the study may limit generalizability; however, the uniformity of care at a specialized center also minimizes heterogeneity due to variation in clinical practice. Second, retrospective design constraints, such as incomplete data on dosing, may affect internal validity. Yet, because renal and liver function markers or PS were adjusted for in our multivariable model, the confounding effect of dosing variability is likely minimal.

In summary, serum sodium and BUN levels may be early, actionable indicators for UAC risk in patients treated with cytotoxic agents; meanwhile, renal function metrics such as eGFR and serum creatinine values may be particularly relevant for those on molecular-targeted drugs. These markers may serve as surrogate endpoints for clinical risk and should be integrated into routine pre-treatment assessments. Future pharmacokinetic studies are warranted to further clarify how renal dysfunction modulates drug metabolism and toxicity in targeted therapies. Tailored supportive care based on drug class and patient profile may ultimately improve patient safety and reduce avoidable acute care needs.

## Supplementary Information

Below is the link to the electronic supplementary material.

Supplementary Material 1

## Data Availability

All data generated or analyzed during this study are included in this published article and its supplementary information files. Restrictions apply to the availability of these data, which were used under license for the current study and are therefore not publicly available. However, de-identified data may be available from the corresponding author upon reasonable request and with permission from the National Cancer Center Hospital East.
