# Supplementary material for: Predictors of chemotherapy-related unplanned acute care in outpatients receiving oral anticancer drugs
Source: Sci Rep. 2025 Oct 6;15:34774. doi: 10.1038/s41598-025-18498-6 (PMC12501367; doi:10.1038/s41598-025-18498-6)
Supplement: Supplementary file 1 — Supplementary Material 1 [file 41598_2025_18498_MOESM1_ESM.docx]

| **Drugs** | **2020** | | | | **2015** | | | |  |
| --- | --- | --- | --- | --- | --- | --- | --- | --- | --- |
|  | **Case group (n=193)** | | **Control group (n=574)** | | **Case group (n=95)** | | **Control group (n=95)** | |  |
|  | **n** | **%** | **n** | **%** | **n** | **%** | **n** | **%** |  |
| **Cytotoxic anticancer drugs** |  |  |  |  |  |  |  |  |  |
| **S-1** | **67** | **34.7** | **217** | **37.8** | **47** | **49.5** | **47** | **49.5** |  |
| **Capecitabine** | **34** | **17.6** | **107** | **18.6** | **10** | **10.5** | **10** | **10.5** |  |
| **Trifluridine/tipiracil** | **17** | **8.8** | **54** | **9.4** | **7** | **7.4** | **7** | **7.4** |  |
| **Tegafur/Uracil** | **-** | **-** | **-** | **-** | **1** | **1.1** | **1** | **1.1** |  |
| **Molecular targeted drugs** |  |  |  |  |  |  |  |  |  |
| **Osimertinib** | **29** | **15** | **91** | **15.9** | **-** | **-** | **-** | **-** |  |
| **Lenvatinib** | **10** | **5.2** | **30** | **5.2** | **1** | **1.1** | **1** | **1.1** |  |
| **Regorafenib** | **8** | **4.1** | **24** | **4.2** | **8** | **8.4** | **8** | **8.4** |  |
| **Palbociclib** | **6** | **3.1** | **13** | **2.3** | **-** | **-** | **-** | **-** |  |
| **Everolimus** | **5** | **2.6** | **10** | **1.7** | **1** | **1.1** | **1** | **1.1** |  |
| **Afatinib** | **4** | **2.1** | **5** | **0.9** | **4** | **4.2** | **4** | **4.2** |  |
| **Alectinib** | **3** | **1.6** | **7** | **1.2** | **-** | **-** | **-** | **-** |  |
| **Abemaciclib** | **3** | **1.6** | **5** | **0.9** | **-** | **-** | **-** | **-** |  |
| **Olaparib** | **2** | **1** | **3** | **0.5** | **-** | **-** | **-** | **-** |  |
| **Trametinib** | **1** | **0.5** | **2** | **0.3** | **-** | **-** | **-** | **-** |  |
| **Sunitinib** | **1** | **0.5** | **2** | **0.3** | **1** | **1.1** | **1** | **1.1** |  |
| **Pazopanib** | **1** | **0.5** | **2** | **0.3** | **1** | **1.1** | **1** | **1.1** |  |
| **Crizotinib** | **1** | **0.5** | **1** | **0.2** | **-** | **-** | **-** | **-** |  |
| **Imatinib** | **1** | **0.5** | **1** | **0.2** | **3** | **3.2** | **3** | **3.2** |  |
| **Gefitinib** | **-** | **-** | **-** | **-** | **4** | **4.2** | **4** | **4.2** |  |
| **Axitinib** | **-** | **-** | **-** | **-** | **3** | **3.2** | **3** | **3.2** |  |
| **Erlotinib** | **-** | **-** | **-** | **-** | **2** | **2.1** | **2** | **2.1** |  |
| A descriptive comparison of drugs commonly used in outpatient cancer chemotherapy between 2015 and 2020.  S-1: Tegafur/Gimeracil/Oteracil | | | | | | | | | |

Supplementary Table S1. Comparison of Drugs Used in 2015 and 2020


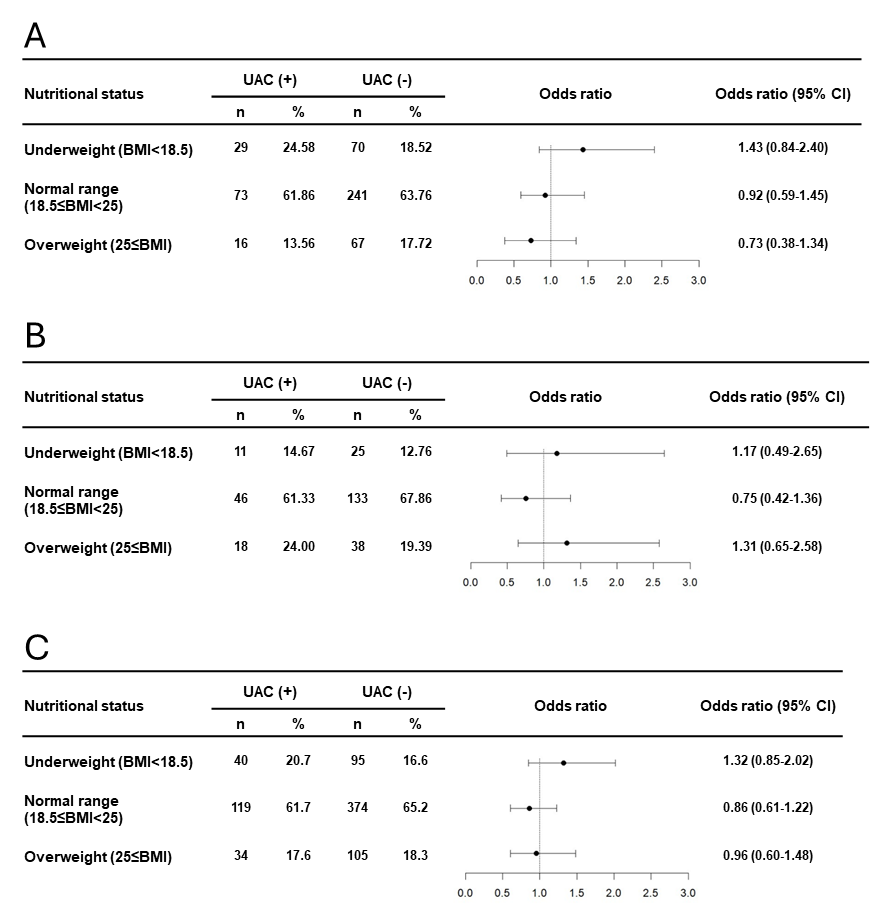


Supplementary Figure S1. Forest Plot of Odds Ratios for UAC Risk Based on BMI

This figure illustrates the classification of BMI into three groups based on nutritional status: “Underweight” (BMI <18.5 kg/m^2^), “Normal range” (18.5 kg/m^2^ ≤ BMI <25 kg/m^2^), and “Overweight” (BMI ≥25 kg/m^2^). Crude odds ratios for UAC occurrence were calculated by comparing each BMI group with the other two groups. Specifically, “Underweight” was compared with the combined “Normal range” and “Overweight” groups, “Normal range” was compared with the combined “Underweight” and “Overweight” groups, and “Overweight” was compared with the combined “Underweight” and “Normal range” groups. The reference group for each comparison comprised the two other categories. Panel (A) shows results for cytotoxic drug users, (B) for molecular-targeted drug users, and (C) for all oral anticancer drug users.

UAC, unplanned acute care; CI, confidence interval; BMI, body mass index
